# Nucleophosmin 1 cooperates with BRD4 to facilitate *c-Myc* transcription to promote prostate cancer progression

**DOI:** 10.1038/s41420-023-01682-w

**Published:** 2023-10-24

**Authors:** Zhe Hong, Chengdang Xu, Shengfeng Zheng, Xinan Wang, Yiran Tao, Yao Tan, Guowen Lin, Denglong Wu, Dingwei Ye

**Affiliations:** 1 Department of Urology, Fudan University Shanghai Cancer Center, 200032 Shanghai, China; 2 Department of Oncology, Shanghai Medical College, Fudan University, 200032 Shanghai, China; 3 Shanghai Genitourinary Cancer Institute, 200032 Shanghai, China; 4 Department of Urology, Tongji Hospital, School of Medicine, Tongji University, 200065 Shanghai, China; 5 Department of Urology, Taizhou Hospital of Zhejiang Province affiliated to Wenzhou Medical University, 317000 Taizhou, China

**Keywords:** Prostate cancer, Oncogenes

## Abstract

Nucleophosmin 1 (NPM1) is a multifunctional protein that promotes tumor progression in various cancers and is associated with a poor prognosis of prostate cancer (PCa). However, the mechanism by which NPM1 exerts its malignant potential in PCa remains elusive. Here, we showed that NPM1 is overexpressed in PCa cell lines and tissues and that the dysregulation of NPM1 promotes PCa proliferation. We also demonstrated that NPM1 transcriptionally upregulates c-Myc expression in PCa cells that is diminished by blockade of bromodomain-containing protein 4 (BRD4). Furthermore, we detected a correlation between NPM1 and c-Myc in patient PCa specimens. Mechanistically, NPM1 influences and cooperates with BRD4 to facilitate *c-Myc* transcription to promote PCa progression. In addition, JQ1, a bromodomain and extra-terminal domain (BET) inhibitor, in combination with NPM1 inhibition suppresses PCa progression in vitro and in vivo. These results indicate that NPM1 promotes PCa progression through a c-Myc **-**mediated pathway via BRD4, and blockade of the NPM1–c-Myc oncogenic pathway may be a therapeutic strategy for PCa.

## Introduction

Prostate cancer (PCa) is the most common type of malignant tumor diagnosed among men in the western world [1, 2]. PCa incidence is also rapidly increasing in Asian countries because of the prostate-specific antigen (PSA) screening project in the elderly population [3]. Although standard androgen deprivation therapy (ADT) works at an early stage, approximately 24 months after treatment with ADT, PCa unavoidably progresses into castration-resistant prostate cancer (CRPC) [4, 5]. The median survival time of patients with CRPC is often less than 20 months once progression to CRPC occurs [6, 7]. Therefore, it is urgent to further study the mechanism underlying PCa progression and identify new therapeutic targets and agents for PCa, especially CRPC.

Nucleophosmin 1 (NPM1) is a multifunctional protein encoded by the *NPM1* gene that is involved in cell proliferation, cell apoptosis, protein chaperoning and other important cellular processes [8, 9]. It is reported that the expression level in colorectal cancer is correlated with tumor sensitivity to chemotherapy and that NPM1 inhibition suppresses colorectal cancer progression by activating p53 and inhibiting protein kinase B (AKT) [10]. Notably, a recently published study showed that NPM1 could regulate the expression of programmed cell death-ligand 1 (PD-L1) at the transcriptional level and promote breast cancer progression [11]. However, the role of NPM1 in PCa and the mechanism by which NPM1 exerts a malignant potential in PCa remain elusive.

The *MYC* proto-oncogene encodes several oncoproteins that participate in transcriptional regulation, apoptosis, cell metabolism, cell cycle progression, and malignant transformation [12, 13]. As a key member of these oncoproteins, c-Myc is often highly expressed in a variety of human cancers and promotes tumor initiation and progression [13]. Although c-Myc overexpression leads to PCa progression mainly through its transcriptional effect [14], the mechanism of c-Myc regulation in PCa and how to leverage this regulation are worth exploring.

In this study, we first tested NPM1 expression in PCa cell lines and tissues and the effect of NPM1 expression on the proliferation and invasion of PCa cells. Second, we conducted an in-depth investigation on the underlying mechanism of NPM1 in cancer cell progression and its potential relationship with c-Myc. Finally, we explored the relationship between bromodomain-containing protein 4 (BRD4) and NPM1 and the effect of JQ1, a bromodomain and extra-terminal domain (BET) inhibitor, on the NPM1–c-Myc oncogenic pathway in vitro and in vivo. Our results demonstrate a novel mechanism wherein that NPM1 cooperates with BRD4 to facilitate *c-Myc* transcription to promote PCa progression. Blockade of the NPM1–c-Myc oncogenic pathway by the BET inhibitor JQ1 may suppress PCa progression, suggesting that NPM1 may be a potential target for PCa treatment.

## Results

### NPM1 is overexpressed in PCa cell lines and tissues

**Fig. 1:**
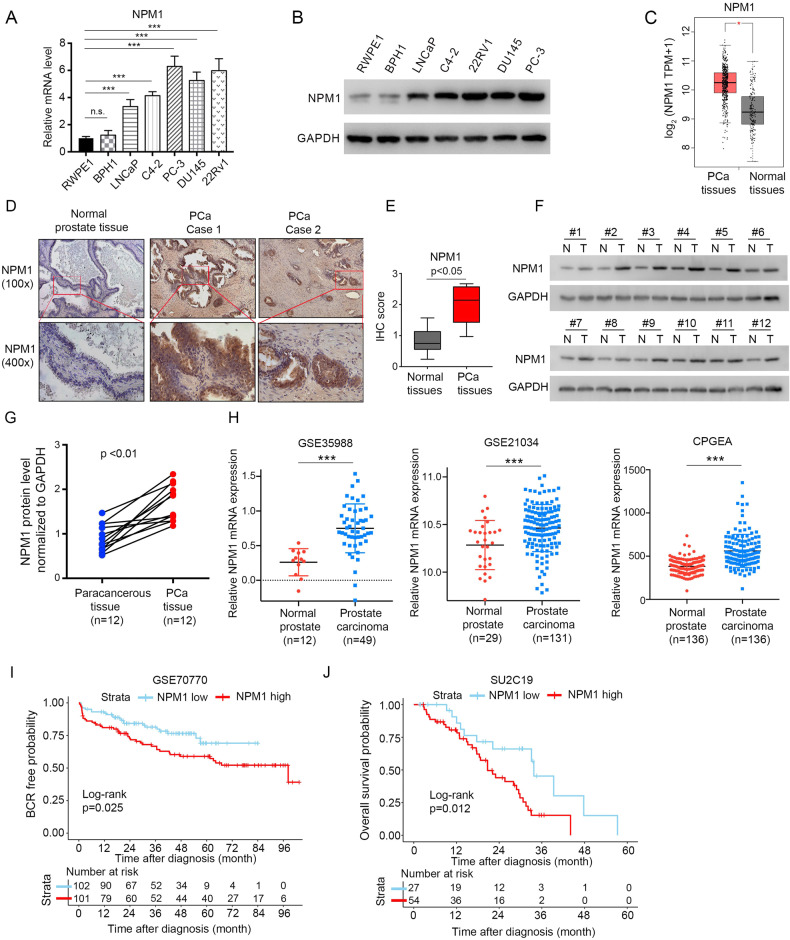
NPM1 is overexpressed in PCa cells and tissues. **A**, **B** The mRNA and protein expression of NPM1 in the most commonly used PCa cell lines, the androgen-responsive cell lines LNCaP and 22Rv1, androgen-independent cell lines C4-2, PC-3, and DU145, and the benign prostate cell lines RWPE1 and BPH1. **C** The mRNA expression level of NPM1 between PCa tissues and normal prostate tissues from TCGA database was analyzed using the GEPIA web tool. Asterisks indicate differences. **D** Representative images for IHC staining of NPM1 protein expression in normal prostate tissues and PCa tissues. Scale bars are shown as indicated. **E** Boxplot of NPM1 expression in normal prostate tissue sections and PCa tissue sections, as determined by IHC scores (normal prostate tissues, *n* = 21; PCa tissues, *n* = 28). The *p* value is shown as indicated. **F**, **G** Western blot analysis of NPM1 expression in 12 paired PCa tissues (T) and matched paracancerous non-tumor tissues (N) from the same patient. GAPDH was used as a loading control, and the protein levels of NPM1 in (**F**) were quantified with ImageJ software and are shown (**G**). **H** The transcriptional levels of NPM1 in the GSE35988, GSE21034 and CPGEA cohorts are shown. **I, J** Relationship between NPM1 expression and biochemical recurrence (BCR)-free survival probability (**I**) as well as overall survival (OS) probability (**J**) was revealed by Kaplan–Meier analysis. Each bar represents the mean values ± SD of three independent experiments except where stated. Statistical analysis was performed using one-way ANOVA with Tukey’s multiple comparisons test for (**A**), two-sided Student’s *t* test for (**E**, **G**), or log-rank test for (**I**, **J**). *p < 0.05; ***p < 0.001; n.s., not significant.

The role of NPM1 in cancer cells, especially PCa cells, remains elusive. We explored NPM1 expression in multiple PCa cell lines. We first tested the mRNA and protein expression of NPM1 in the most commonly used PCa cell lines, the androgen-responsive cell lines LNCaP and 22Rv1, androgen-independent cell lines C4-2, PC-3, and DU145, and the benign prostate cell lines RWPE1 and BPH1. Our data showed that NPM1 expression in PCa cells was higher than that in benign prostate cells (Fig. 1A, B), and the expression was consistent with the increase in malignancy grade. We also checked NPM1 expression in PCa tissues and prostate non-cancer tissues using the gene expression profiling interactive analysis (GEPIA) web tool (http://gepia2.cancer-pku.cn/#index). Consistently, we observed that NPM1 expression in PCa tissues was higher than that in prostate non-cancer tissues (Fig. 1C).

To verify these findings, we measured the NPM1 protein level in the PCa surgery specimens by immunohistochemical (IHC) staining of PCa tissues (*n* = 28) and noncancerous counterparts (*n* = 21). IHC results were evaluated by assessing the staining intensity, and representative images are shown in Fig. 1D. The quantified data are shown in Fig. 1E, which demonstrates that NPM1 expression was increased in PCa tissues compared to that in normal (non-tumor) tissues. Furthermore, the NPM1 level in cancer tissues and paired noncancerous tissues of 12 patients was tested, and the results revealed that NPM1 expression was significantly increased in cancer tissues (Fig. 1F, G). We then investigated NPM1 expression in silico, and the results demonstrated that NPM1 was more highly expressed in the PCa cohorts (GSE21034 and GSE35988) downloaded from the GEO database (https://www.ncbi.nlm.nih.gov/geo/) than in normal prostate tissues. Accordingly, we observed a similar trend in a PCa cohort based on the Chinese population (Chinese Prostate Cancer Genome and Epigenome Atlas, CPGEA) (http://www.CPGEA.com/) (Fig. 1H). Furthermore, Kaplan–Meier analysis revealed a significant relationship between higher SMYD2 expression and shorter biochemical recurrence (BCR)-free survival probability as well as overall survival (OS) probability (Fig. 1I, J) from the GSE70770 and SU2C19 datasets downloaded from the GEO database (https://www.ncbi.nlm.nih.gov/geo/). Collectively, these data suggest that increased NPM1 expression in PCa is associated with tumorigenesis and poor prognosis.

### NPM1 overexpression leads to the proliferation and invasion of PCa cells

**Fig. 2:**
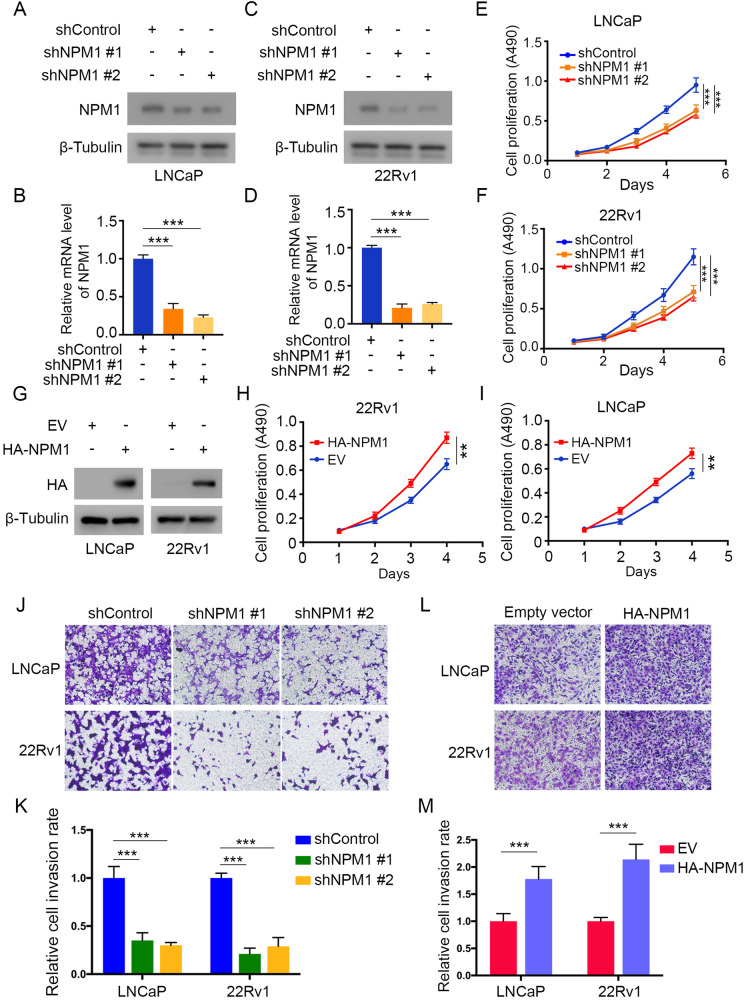
NPM1 overexpression leads to the proliferation and invasion of PCa cells. **A**, **B** LNCaP cells were infected with lentiviral vectors expressing control or NPM1-specific shRNAs for 48 h and selected with puromycin (10 μg/ml) for another 72 h. Then, the cells were harvested for western blot (**A**) and qRT‒PCR (**B**) analyses. **C**, **D** 22Rv1 cells were infected with lentiviral vectors expressing control or NPM1-specific shRNAs for 48 h and selected with puromycin (10 μg/ml) for another 72 h. Then, the cells were harvested for western blot (**C**) and qRT-PCR (**D**) analyses. **E**, **F** The proliferation of LNCaP and 22Rv1 cells infected with the indicated shRNA was measured by MTS assays. **G**‒**I** LNCaP and 22Rv1 cells were transfected with the indicated plasmids for 48 h and collected for western blot analysis. Cell proliferation rates were measured by MTS assays. **J**, **K** LNCaP and 22Rv1 cells were infected with lentiviral vectors expressing control or NPM1-specific shRNAs for 48 h and selected with puromycin (10 μg/ml) for another 72 h. Then, the cells were used for Transwell assays (**J**), and the quantitative data are shown (**K**). **L**, **M** LNCaP and 22Rv1 cells were transfected with the indicated plasmids for 48 h and were then used for Transwell assays (**L**), and the quantitative data are shown (**M**). Each bar represents the mean values ± SD of three independent experiments. Statistical analysis was performed using one-way ANOVA with Tukey’s multiple comparisons test for (**B**, **D**–**F**, **K**), and two-sided Student’s *t* test for (**H**, **I**, **M**). **p < 0.01; ***p < 0.001.

Since NPM1 expression is increased in PCa cells and tissues, we explored the biological function of NPM1 expression in PCa. First, we knocked down NPM1 expression by a pair of short hairpin RNAs (shRNAs) in LNCaP (Fig. 2A, B) and 22Rv1 cells (Fig. 2C, D) and found by utilizing MTS assays that cell proliferation was dramatically impaired when NPM1 was knocked down (Fig. 2E, F). Conversely, when NPM1 was overexpressed in 22Rv1 and LNCaP cells by ectopically transfecting HA-tagged NPM1 plasmids (Fig. 2G), we detected accelerated growth of PCa cells (Fig. 2H, I). We further tested the cancer cell invasion ability by Transwell assays, and the results revealed that NPM1 upregulation was positively correlated with the invasion ability of PCa cells. Knockdown of NPM1 resulted in decreased invasion ability in both the LNCaP and 22Rv1 cell lines (Fig. 2J, K), while overexpression of NPM1 increased the cell invasion ability (Fig. 2L, M). Taken together, these data revealed that NPM1 is involved in PCa tumorigenesis by regulating cell proliferation and invasion.

### C-Myc is a downstream target of NPM1 in PCa cells

**Fig. 3:**
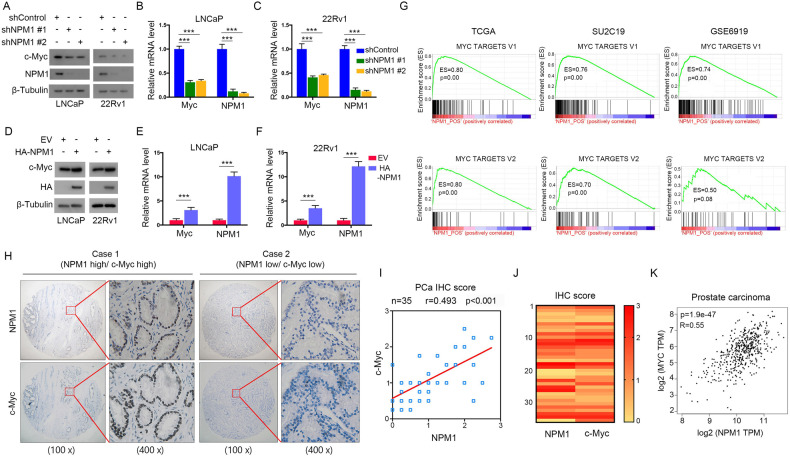
C-Myc is a downstream target of NPM1 in PCa cells. **A**‒**C** LNCaP and 22Rv1 cells were infected with lentiviral vectors expressing control or NPM1-specific shRNAs for 48 h and selected with puromycin (10 μg/ml) for another 72 h. Then, the cells were harvested for western blot (**A**) and qRT‒PCR (**B**, **C**) analyses. **D**‒**F** LNCaP and 22Rv1 cells were transfected with the indicated plasmids for 48 h and collected for western blot (**D**) and qRT-PCR (**E**, **F**) analyses. **G** GSEA indicated that NPM1 expression was related to MYC pathway in PCa. **H** Images of NPM1 and c-Myc for IHC staining in PCa tissue sections. Scale bars are shown as the indicated. **I**, **J** The correlation (**I**) and heatmap (**J**) analyses between NPM1 and c-Myc from the IHC staining scores in (**H**) are shown. **K** The correlation between NPM1 and MYC mRNA expressions in patient PCa samples was determined by the GEPIA webtool. Each bar represents the mean values ± SD of three independent experiments. Statistical analysis was performed using one-way ANOVA with Tukey’s multiple comparisons test for (**B**, **C**), two-sided Student’s *t* test for (**E**, **F**), or Spearman’s correlation analysis for (**I**, **K**). ***p < 0.001.

The above data showed that NPM1 not only is highly expressed in PCa tissues but also facilitates PCa progression by modulating cancer cell proliferation and invasion. However, the underlying molecular mechanism remains unclear. Interestingly, we found that c-Myc expression was decreased in NPM1 knocked down LNCaP and 22Rv1 cells (Fig. 3A‒C). In line with this finding, the expression levels of c-Myc increased when PCa cells ectopically expressed NPM1 (Fig. 3D‒F). To investigate the underlying mechanism by which targeting NPM1 activates PCa progression, GSEA method was used to identify the possible pathway affected by NPM1. In the three independent open access PCa data sets (TCGA, SU2C19, GSE6919), the GSEA results showed that high NPM1 expression resulted in significant enrichment of the MYC pathway (Fig. 3G) (https://www.gsea-msigdb.org/). To elucidate the correlation between these two genes in PCa cells, we performed IHC staining in 28 patient specimens, and the images of NPM1 and c-Myc staining are shown (Fig. 3H). We observed a positive correlation between NPM1 and c-Myc from the IHC staining scores in patient samples (Fig. 3I, J). We further checked the correlation between the two genes in silico, and the result demonstrated a significant positive correlation with a *p* value < 0.001 (Fig. 3K). Such a correlation was also observed in multiple cancer types, including breast adenocarcinoma, pancreatic adenocarcinoma, stomach adenocarcinoma, lung carcinoma, thyroid carcinoma, colon carcinoma and cervical carcinoma (Fig. S1). Collectively, these data indicated that c-Myc is regulated by NPM1 and is a downstream target of NPM1.

### The oncogenic function of NPM1 in PCa is performed via a c-Myc-mediated pathway

**Fig. 4:**
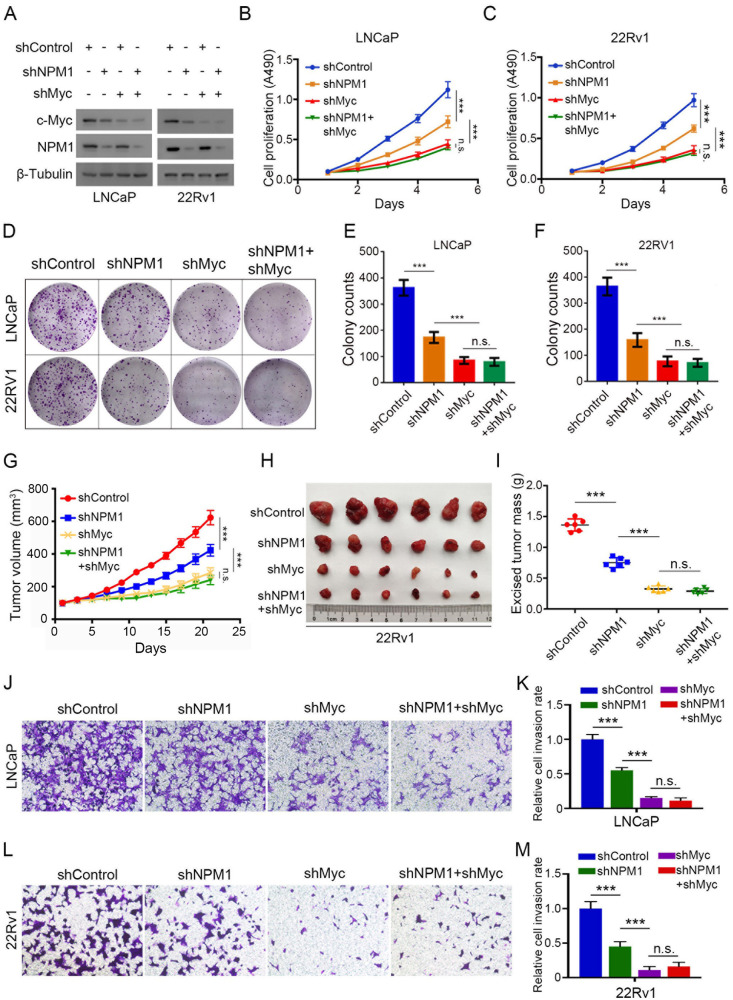
The oncogenic function of NPM1 in PCa is performed through a c-Myc-mediated pathway. **A**‒**C** LNCaP and 22Rv1 cells were infected with lentiviral vectors expressing control or NPM1-specific shRNAs for 48 h and selected with puromycin (10 μg/ml) for another 72 h. Then, the cells were used for western blot (**A**) analysis and MTS assays (**B**, **C**). **D**‒**F** LNCaP and 22Rv1 cells were infected with lentiviral vectors expressing control or NPM1-specific shRNAs for 48 h and selected with puromycin (10 μg/ml) for another 72 h. Then, the cells were used for colony formation assays (**D**). The quantitative data are shown in (**E**, **F**). **G**‒**I** 22Rv1 cells were infected with lentiviral vectors expressing control or NPM1-specific shRNAs for 48 h and selected with puromycin (10 μg/ml) for another 72 h. Then, the cells were injected subcutaneously into the NOD/SCID mice. Mice were observed for 21 days and sacrificed, tumor growth curves were constructed (**G**), tumors were collected and photographed (**H**), and tumor weights were measured (**I**) (*n* = 6 mice/group). **J**‒**M** LNCaP and 22Rv1 cells were infected with lentiviral vectors expressing the indicated shRNAs for 48 h and selected with puromycin (10 μg/ml) for another 72 h. Then, the cells were used for Transwell assays (**J**, **L**), and the quantitative data are shown (**K**, **M**). Each bar represents the mean values ± SD of three independent experiments except where stated. Statistical analysis was performed using one-way ANOVA with Tukey’s multiple comparisons test for (**B**, **C**, **E**–**G**, **I**, **K**, **M**). ***p < 0.001; n.s., not significant.

Since c-Myc is regulated by NPM1 in PCa cells, and that *c-Myc* is a well-known oncogene driving multiple cancers, including PCa, we hypothesize that the oncogenic function of NPM1 in PCa is performed through a c-Myc-mediated pathway. To verify this hypothesis, we performed in vitro assays by knocking down NPM1 and c-Myc in LNCaP and 22Rv1 cells (Fig. 4A). MTS assays showed that single knockdown of NPM1 drastically reduced the growth rate of LNCaP and 22Rv1 cells, while single knockdown of c-Myc had a more prominent impact on cancer cell proliferation. Interestingly, combined with knockdown of NPM1 and c-Myc, the cell proliferation rates did not decrease further (Fig. 4B, C). Similar trends were observed in colony formation assays (Fig. 4D‒F). Furthermore, the results in the mouse xenograft models confirmed the observation that knockdown of NPM1 or c-Myc alone reduced tumor growth, but double knockdown of NPM1 and c-Myc did not decrease the tumor growth rate more than single knockdown of c-Myc (Fig. 4G‒I). The invasion ability of PCa cells was also evaluated, and the results demonstrated that single knockdown of NPM1 or c-Myc reduced the cell invasion ability, as expected, whereas compared with single c-Myc knockdown, double knockdown of NPM1 and c-Myc did not further reduce the invasion rate (Fig. 4J‒M). Collectively, these results suggested that the oncogenic function of NPM1 in PCa is mainly mediated by c-Myc.

### BRD4 is involved in the NPM1–c-Myc oncogenic pathway

**Fig. 5:**
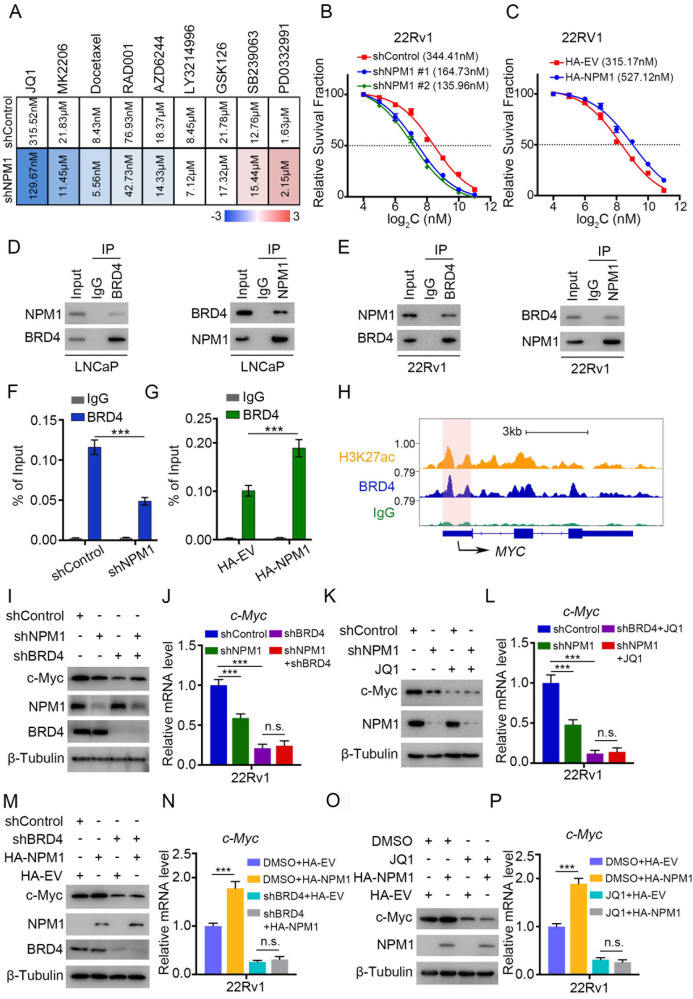
BRD4 is involved in the NPM1‒c-Myc oncogenic pathway. **A** 22Rv1 cells were infected with lentiviral vectors expressing control or NPM1-specific shRNAs for 48 h and selected with puromycin (10 μg/ml) for another 72 h. Then, the cells were treated with the different type of small inhibitors for 48 h, an PARP1IC50) were analyzed and shown in the heatmap. The shNPM1 indicated that the mixed shRNA of shNPM1#1 and shNPM1#2. **B**, **C** The dose-effect relationship curves of JQ1 in 22Rv1 cells. 22Rv1 cells were infected with lentiviral vectors expressing control or NPM1-specific shRNAs, respectively, and treated with the different concentrations of JQ1 (**B**). 22Rv1 cells were transfected with HA-EV and HA-NPM1, respectively, and treated with the different concentrations of JQ1 (**C**). **D**, **E** 293 T cells transfected with the indicated plasmids were harvested for co-immunoprecipitation assays. **F**, **G** The ChIP‒qPCR for BRD4 in 22rv1 cells. 22Rv1 cells were infected with lentiviral vectors expressing control or NPM1-specific shRNAs for 48 h and selected with puromycin (10 μg/ml) for another 72 h. Then, the cells were harvested for ChIP‒qPCR analysis (**F**). 22Rv1 cells were transfected with HA-EV and HA-NPM1, respectively. Then, the cells were harvested for ChIP‒qPCR analysis (**G**). **H** The condition of BRD4 binding to the *MYC* promoter in the UCSC Genome-Browser ChIP-sequence data. **I**, **J** 22Rv1 cells were infected with lentiviral vectors expressing control or NPM1-specific shRNAs or BRD4-specific shRNAs for 48 h and selected with puromycin (10 μg/ml) for another 72 h. Then, the cells were harvested for western blot (**I**) and qRT‒PCR (**J**) analyses. **K**, **L** 22Rv1 cells were infected with lentiviral vectors expressing control or NPM1-specific shRNAs for 48 h and selected with puromycin (10 μg/ml) for another 72 h. Then, the cells were treated with or without JQ1 (3 μM) for 24 h and harvested for western blot (**K**) and qRT‒PCR (**L**) analyses. **M**, **N** 22Rv1 cells were infected with lentiviral vectors expressing control or BRD4-specific shRNAs and transfected with the indicated plasmids. Then, the cells were harvested for western blot (**M**) and qRT‒PCR (N) analyses. **O**, **P** 22Rv1 cells were transfected with the indicated plasmids, then treated with or without JQ1 (3 μM) for 24 h and harvested for western blot (**O**) and qRT‒PCR (**P**) analyses. Each bar represents the mean values ± SD of three independent experiments. Statistical analysis was performed using two-sided Student’s *t* test for (**F**, **G**, **N**, **P**) and one-way ANOVA with Tukey’s multiple comparisons test for (**J**, **L**). ***p < 0.001; n.s., not significant.

To explore the potential therapeutic significance of NPM1 in PCa, especially CRPC, we tested and compared the sensitivity of several small molecule drugs on shControl and shNPM1 22Rv1 cells. As shown in Fig. 5A, we discovered that PCa cells were more sensitive to treatment with some small molecule drugs (JQ1, MK2206, docetaxcel, RAD001 and AZD6244) when NPM1was knocked down, while their sensitive to some drugs (LY3214996 and GSK126) changed little, and their resistance to some drugs (SB239063 and PD0332991) was even increased. Among these drugs, JQ1, a bromodomain and extra-terminal domain (BET) inhibitor, showed the most remarked reduction in the IC50 from 315.52 nM to 129.67 nM (Fig. 5A), which prompted us to hypothesize that BET family members may be involved in this process.

NPM1 overexpression reduced the sensitivity of 22Rv1 cells to JQ1 treatment, whereas NPM1 silencing increased the sensitivity of 22Rv1 cells to JQ1 (the IC50 of JQ1 decreased), as expected (Fig. 5B, C). As bromodomain containing 4 (BRD4) is a well-known and effective BET protein and is well acknowledged in cancers for its oncogenic roles, we tested the relationship of NPM1 and BRD4. The coimmunoprecipitation assay showed that there was a strong endogenous interaction between NPM1 and BRD4 in both PCa and CRPC cells. BRD4 acts as a transcriptional and epigenetic regulator by serving as a reader of acetylated lysine residues on histones. As shown in our previously published data [15–17], BRD4 can promote gene transcription by taking advantage of histone acetyltransferase 1 (HAT1) [16]. It has been reported that BRD4 binds to the P-TEFb complex [18], which is crucial for transcription elongation. Thus, we proposed that NPM1 may influence and cooperate with BRD4 to facilitate downstream target transcription. We already showed that c-Myc may serve as a potential downstream gene of NPM1 (Fig. 3A‒F). Hence, we performed a ChIP (chromatin immunoprecipitation)-qPCR assay with NPM1 knockdown or overexpression. The data demonstrated that the binding of BRD4 to c-Myc was decreased when NPM1 knockdown, whereas the binding increased when NPM1 overexpression (Fig. 5D‒G).

Moreover, we assessed the ChIP-seq data and observed a binding peak in the promoter region of *Myc* (Fig. 5H). These data indicated that both NPM1 and BRD4 are involved in c-Myc regulation. To further address this observation, we generated stable cell lines with single knockdown of NPM1 or BRD4 as well as double knockdown of NPM1 and BRD4. We showed that silencing NPM1 or BRD4 alone reduced the expression of c-Myc, while dual silencing did not further attenuate c-Myc expression (Fig. 5I, J). A similar tendency was observed in both the presence and absence of JQ1 treatment (Fig. 5K, L). In contrast, overexpression of NPM1 increased the expression of c-Myc, but this trend was moderated in the context of BRD4 blockade (Fig. 5M‒P). Collectively, these data illustrated that NPM1 could epigenetically regulate c-Myc expression, and that this regulatory mechanism occurs in a BRD4-mediated manner.

### BET inhibitor blocks the NPM1–c-Myc oncogenic pathway to suppress PCa progression in vitro and in vivo

**Fig. 6:**
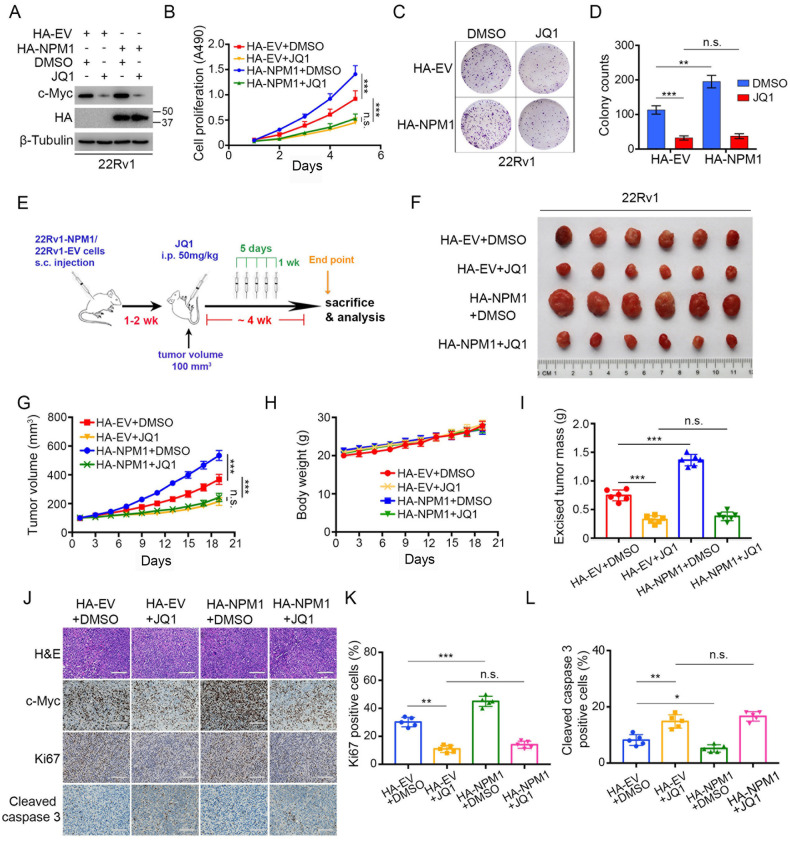
BET inhibitor blocks the NPM1‒c-Myc oncogenic pathway to suppress PCa progression in vitro and in vivo. **A**‒**D** 22Rv1 cells were transfected with the indicated plasmids. Then, the cells were treated with or without JQ1 (3 μM) for 24 h and harvested for western blot analysis (**A**), MTS assays (**B**) and colony formation assays (**C**). The quantitative data of (**C**) are shown (**D**). **E** The schematic diagram demonstrating the drug treatment method for mice bearing subcutaneous tumors. **F**‒**I** 22Rv1 cells from (**A**) were mixed with Matrigel and injected subcutaneously (s.c.) into the right dorsal flanks of NOD/SCID mice (*n* = 6 mice/group) and then treated with JQ1 (50 mg/kg, i.p.) for 5 days during one week when the average size of tumors reached 100 mm^3^. The tumors were harvested on Day 21 and photographed (**F**), and the growth curves of tumors were measured (**G**). The mice (**H**) and tumors (**I**) were weighted. **J** H&E and IHC staining for c-Myc, Ki-67 and cleaved caspase 3 were performed. Representative images were taken from each group. Scale bars = 50 μm. **K**, **L** Ki-67 (**K**) and cleaved caspase 3 (**L**) positive cells in the tissue sections from (**J**) were quantified. The number of positive cells from at least five fields was counted and analyzed (**K**, **L**). Each bar represents the mean values ± SD of three independent experiments except where stated. Statistical analysis was performed using one-way ANOVA with Tukey’s multiple comparisons test for (**B**, **D**, **G**–**I**, **K**, **L**). *p < 0.05; **p < 0.01; ***p < 0.001; n.s., not significant.

Given that BRD4 is involved in NPM1-mediated oncogenic function, we investigated whether the BET inhibitor JQ1 can block the detrimental effects of NPM1 in cells and mouse models. We transfected HA-tagged empty vector (EV) and NPM1 plasmids into 22Rv1 cells and treated them with or without JQ1 (Fig. 6A). MTS and colony formation assays demonstrated that JQ1 markedly reduced cell growth and colony numbers in both the HA-EV and HA-NPM1 groups (Fig. 6B‒D). Interestingly, we did not observed difference between the HA-EV and HA-NPM1 groups when treated with JQ1, which is consistent with the previous result that NPM1 regulated cancer cell growth through BRD4 (Fig. 5K, L, O, P). Furthermore, we performed in vivo assays by using NOD/SCID mice. JQ1 was injected intraperitoneally at a dosage of 50 mg/kg for 4 weeks (Fig. 6E). As demonstrated in Fig. 6F‒I, JQ1 treatment effectively curbed tumor growth in vivo and abolished the growth-promoting effects of NPM1. The tumor samples were excised and embedded in paraffin and were then subjected to IHC staining for c-Myc, Ki67 and cleaved caspase 3 (Fig. 6J). Quantitative data are shown in Fig. 6K, L. Taken together, these data showed that treatment with a BET inhibitor can block the NPM1–c-Myc oncogenic pathway to suppress PCa progression.

## Discussion

In this study, we showed that NPM1 is overexpressed in PCa cell lines and PCa tissues and that the dysregulation of NPM1 promotes the cell proliferation and invasion of PCa. Importantly, c-Myc is transcriptionally upregulated by NPM1 via BRD4, which is required for the NPM1-induced PCa progression. By contrast, BET inhibitor JQ1 blocks the NPM1–c-Myc oncogenic pathway, thereby inhibits PCa progression in vitro and in vivo. Our results suggest a novel mechanism by which NPM1 promotes PCa progression through a c-Myc -mediated pathway via BRD4.

NPM1 has been reported to epigenetically regulate PD-L1 expression in breast cancer, and once bound to poly (ADP-ribose) polymerase-1 (PARP1), NPM1 loses the ability to activate PD-L1 [11]. In addition, NPM1 expression has also been shown to be correlated with prognosis in colorectal cancer, and targeting NPM1 could inhibit the progression of colorectal cancer by activating p53 and inhibiting AKT [10, 19]. In the urogenital system, NPM1 could promote cell proliferation and migration and drug resistance in bladder cancer [20]. In this study, we uncovered that NPM1 was aberrantly overexpressed in PCa cells, suggesting that NPM1 may have a vicious role in PCa cells. Knockdown of NPM1 reduced the growth rate and invasion ability of PCa cells, whereas increasing NPM1 expression accelerated cancer cell proliferation and invasion. Our data clearly show that NPM1 also exerts its carcinogenic function and role in PCa, which prompts us to further examine the detailed underlying mechanisms.

There are some potential therapeutic targets in PCa, including AR, c-Myc, AKT, and recombinant forkhead box protein A1 (FOXA1) [21–24], among which AR has attracted considerable research attention, but the role of c-Myc oncoprotein in PCa remains little studied. In this study, we noted that the expression of c-Myc decreased when NPM1 was knocked down, whereas ectopic expression of NPM1 increased c-Myc expression in PCa, suggesting that the c-Myc oncoprotein mediates the carcinogenic effect of NPM1 in PCa. In fact, as an oncoprotein, c-Myc is frequently highly expressed in human cancers and exerts carcinogenic effects to promote tumor initiation and progression [13]. As a transcription factor, c-Myc binds and initiates the transcription of specific target oncogenes, including *AR* [25]. All these findings clearly indicate that c-Myc is widely dysregulated in cancer development. However, compared with knowledge about how c-Myc exerts its oncogenic functions, knowledge about how c-Myc is regulated in PCa is limited. Our results demonstrated that c-Myc expression was transcriptionally upregulated by NPM1 in PCa. Since NPM1 could drive PCa progression, we further tested the role of c-Myc in this process. As a result, we found that c-Myc served as a downstream target of NPM1 to mediate the carcinogenic effect of NPM1 in PCa progression.

BRD4 is the most active oncogenic member of the BET family [26–29]. In our previous work, we showed that BRD4, as an epigenetic reader specific for acetylated lysine residues, cooperates together with histone acetyltransferase 1 (HAT1) to regulate AR and AR- variants and further influence sensitivity to enzalutamide [16]. We also identified a demethylase, KDM5C, as a downstream target of BRD4 and uncovered a negative regulatory effect of BRD4 on PTEN [15]. These findings promoted us to investigate the relationship between NPM1 and BRD4. Given that *c-Myc* may serve as a potential downstream gene of NPM1 (Fig. 3) and that the binding of BRD4 to c-Myc was decreased when NPM1 was knocked down but increased when NPM1 was overexpressed in PCa cells (Fig. 5D‒G), we hypothesized that NPM1 may influence and cooperate with BRD4 to facilitate downstream target transcription. Moreover, we observed that single silencing of NPM1 or BRD4 reduced the expression of c-Myc, whereas dual silencing did not further attenuate c-Myc expression (Fig. 5I‒L). Conversely, overexpression of NPM1 increased the expression of c-Myc, but this trend was moderated in the context of BRD4 blockade (Fig. 5M‒P). Taken together, our results showed that NPM1 epigenetically upregulates c-Myc expression and that this regulatory mechanism occurs in a BRD4-mediated manner.

Further, a paper published in *Nature* pointed out the possibility of targeting the BET family in CRPC treatment; this paper said that AR signaling-competent human CRPC cell lines are preferentially sensitive to BET inhibition [30]. As a BRD4 inhibitor, JQ1 has a strong inhibitory effect on a variety of cancers, including PCa [31], and it has shown great application prospects in the treatment of various cancers. However, some cancer centers have already shown resistance to JQ1 treatment in the laboratory and clinic due to the lack of an elaborate understanding of the mechanism of JQ1 [32–35]. In our study, we found that JQ1 could block the oncogenic NPM1–c-Myc pathway in vitro and in vivo, which further verified that NPM1 promotes PCa progression by BRD4 mediation and provided more evidence for the future application of JQ1 in PCa patients.

Although our results clearly demonstrated a novel mechanism suggesting that NPM1 promotes PCa progression through BRD4 via the c-Myc **-**mediated pathway, which can be suppressed by JQ1, a BET inhibitor, there are still some problems to be studied. For example, that NPM1 is physically and/or functionally associated with c-Myc needs to be further clarified. The detailed mechanism by which NPM1 interacts directly with BRD4, such as preventing BRD4 ubiquitination, affecting BRD4 nuclear localization or other pathways to promote its transcriptional activity, also requires to be thoroughly investigated.

**Fig. 7:**
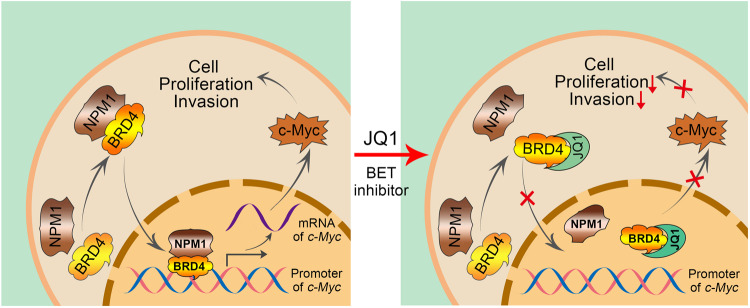
A working model based on the current findings. Left: NPM1 interacts with BRD4 to lead to BRD4 binding to the promoter of *MYC* gene to promote the *MYC* transcription, which in turn initiates the proliferation and invasion of PCa cells. Right: BET inhibitors block the interaction between NPM1 and BRD4 to stop the transcription of *MYC* gene to inhibit the PCa progression.

In summary, NPM1 promotes PCa progression through a c-Myc **-**mediated pathway via BRD4, and the oncogenic function of NPM1 in PCa can be blocked by the BET inhibitor JQ1 (Fig. 7), indicating that NPM1 may be an effective target for PCa treatment and that blockading the NPM1–c-Myc oncogenic pathway may be a therapeutic strategy for PCa.

## Materials and methods

### Cell lines and cell culture conditions

The LNCaP, C4-2, DU145, 22Rv1, PC-3, BPH1, RWPE1 and HEK293T cell lines (ATCC, Manassas, VA) were cultured in RPMI-1640 (PC-3, LNCaP, DU145, C4-2, 22Rv1 and BPH1), DMEM (HEK293T) or K-SFM (RWPE1) medium supplemented with 10% FBS and 1% penicillin–streptomycin in a 5% (v/v) humidified incubator at a steady temperature of 37 °C. All cells have been authenticated using short tandem repeat (STR) profiling. All experiments were performed with mycoplasma-free cells.

### Genetic modulation in PCa cell lines

A lentivirus transduction system was used to generate stable cell lines with specific gene knockdown. The shNPM1 or shControl plasmid, pMD2.G envelope plasmid and psPAX2 packaging plasmid were transfected into HEK293T cells using PEI transfection reagent. The lentiviral supernatant was collected and used to infect the indicated cells for 48 h. Polybrene (10 mg/ml) was added to the cell culture supernatant for 24 h. The HA-NPM1 and HA-EV plasmids were transfected into the indicated cells with the Lipo 2000 transfection reagent for 48 h, and the cells were then retained for further study. The shRNA sequences used in this study were as follows: shNPM1 # 1: (5′- CCGGGCGCCAGTGAAGAAATCTATACTCGAGTATAGATTTCTTCACTGGCGCTTTTTG -3′); shNPM1 # 2: (5′- CCGGGCAAAGGATGAGTTGCACATTCTCGAGAATGTGCAACTCATCCTTTGCTTTTTG -3′); shMyc # 1: (5′- CCGGCCTGAGACAGATCAGCAACAACTCGAGTTGTTGCTGATCTGTCTCAGGTTTTTG-3′); and shMyc # 2: (5′- CCGGTACGGAACTCTTGTGCGTAAGCTCGAGCTTACGCACAAGAGTTCCGTATTTTTG -3′).

### Cell proliferation assay

Cell proliferation assays were performed using a Cell Counting Kit-8 (CCK-8, Beyotime Biotechnology, China) kit. Briefly, 3000 cells/well were dispensed in 100 μl aliquots, seeded in a 96-well plate, and then starved for 72 h. At the indicated time points, 10 μl CCK-8 reagent was added to the cells. After incubation in a 37 °C incubator for 4 h, cell growth was assessed by detecting the OD value at 450 nm wavelength in a microplate reader to estimate the number of live cells in each well. The growth curve was analyzed and plotted using GraphPad Prism® 7.0.

### Cell invasion assay

The invasion capacity of PCa cells was measured using transwell and 3D invasion assays. The upper chamber membrane was precoated with Matrigel (BD Corning, USA) for 4 h before the experiment. Briefly, the cells were plated in the upper chambers at a density of 1 × 10^5^ cells/well. After 48 h of incubation, the non-invaded cells were rinsed gently, and cells invading the bottom of the membranes were fixed with 4% paraformaldehyde and stained with 1% toluidine blue. The cell numbers were counted in three randomized fields.

### Patient PCa tissues specimen

Specimens of patient PCa and paracancerous tissues were obtained from the department of urology of Fudan University Shanghai Cancer Center, with patient consent under a protocol approved by the Ethics Committee of Fudan University Shanghai Cancer Center (050432-4-2108).

### RNA isolation and qRT‒PCR

The cells were starved for at least 48 h after transfection. Total RNA was isolated using a TRIzol reagent (Sigma, USA). An RNA-to-cDNA kit (TaKaRa, Japan) was used for cDNA synthesis by reverse transcription. SYBR Green Mix (TaKaRa, Japan) and a CFX96 Real-Time System were used for RT-PCR. The primers used for qRT**–**PCR were as follows: c-Myc: 5′ -GGCTCCTGGCAAAAGGTCA -3′ (forward) and 5′ -CTGCGTAGTTGTGCTGATGT -3′ (reverse); and NPM1: 5′ -GGTGGTAGCAAGGTTCCACAG -3′ (forward) and 5′ - TTCTTCACTGGCGCTTTTTCT -3′ (reverse).

### Western blotting

Total protein was extracted from the cells with RIPA lysis Buffer containing protease inhibitors (Beyotime, China) and separated on a 10% or 12% SDS-PAGE gel. The proteins were transferred onto nitrocellulose (NC) membranes (Millipore, USA), the NC membranes were blocked with 5% BSA (Sigma-Aldrich, USA) for 1 h at room temperature, and further incubated with primary antibodies at 4 °C overnight. NC membranes were washed three times with TBST (1×) for 10 min each and incubated with the corresponding secondary antibodies for 1 h. After washing thrice with TBST (1×), the membranes were exposed and visualized using an ECL system (Thermo Fisher Scientific, USA). Main antibodies used were listed in Table S1.

### Immunohistochemical (IHC) staining

Patient tissues were fixed with 10% formaldehyde, embedded in 4% paraffin, cut into 4 μm thick slices, dehydrated, and rehydrated in xylene and an ethanol gradient (100% > 95% > 80% > 70%). To enhance antigen exposure, the slices were treated with sodium citrate (pH = 6) at 98 °C for 15 min for antigen retrieval, endogenous peroxidase activity was blocked using 3% hydrogen peroxide in PBS for 15 min. Sections were blocked with 5% BSA for 30 min at room temperature before IHC staining with specific primary antibodies against NPM1 (1:10,000, Abcam) at 4 °C overnight. Detailed information on the protocols used for IHC staining, antigen retrieval, and immunostaining has been previously described [17]. Main antibodies used were listed in Table S1.

### Chromatin immunoprecipitation (ChIP) quantitative PCR (ChIP‒qPCR)

ChIP assay was performed by applying the ChIP Kit (Abcam, ab500) according to manufacturer’s instruction. DNA fragments were purified and analyzed by quantitative-PCR to measure DNA-binding intensity by using the PCR Reagents and Kit (Abcam, ab270816) according to manufacturer’s instruction. The following primer sequences were used for ChIP analyses: MYC-forward: CTCTGGAACAGGCAGACACA; MYC-reverse: TGCACAGCTATCTGGATTGG.

### Mouse xenograft studies

Male NOD/SCID mice (Shanghai SLAC Laboratory Animal Company), 5–7 weeks old, were individually housed in the Animal Center of Fudan University Shanghai Cancer Center, with free access to food and water, at a suitable temperature and humidity, and under light (12-h light/12-h dark).

After 1 week of adaptive feeding, all animals were randomly divided into four groups for cell injection. A total of 2 × 10^6^ 22Rv1 cells combined with Matrigel (1:1, Corning, USA) were transplanted into the right flank of mice as indicated. Mice were included in the study if the transplantation was successful; otherwise, they were excluded until there were 6 mice in each group. The growth of the xenografts in mice was observed and recorded every other day, and the size of the xenografts was measured using calipers. The mice were sacrificed with ether anesthesia on day 21, and the tumors were retained. No blinding was used for the animal experiments. All mouse experiments were ethical and approved by the Institutional Animal Care and Use Committee of Fudan University Shanghai Cancer Center.

### Statistical analysis

All statistical analysis in this study was performed using SPSS 19.0 software (SPSS Inc., USA) and GraphPad Prism 8 (GraphPad Software, USA). Data were collected from at least three independent experiments except where specified, and are expressed as the mean ± standard deviation (SD). The criteria calculating the results were pre-established. All testing and data analysis were conducted in a blinded manner. Samples were randomly allocated to different treatment conditions. Representative areas of cell culture chambers were randomly selected for imaging with the microscope. One-way analysis of variance (ANOVA) (for normally distributed data and multiple groups) was used to analyze the quantitative data, as were the Kruskal–Wallis test (for non-normally distributed data and multiple groups, if yes), the two-sided Student’s *t* test (for normally distributed data between two groups), or the Mann–Whitney *U*-test (for non-normally distributed data between two groups, if yes). Survival curves were constructed using Kaplan–Meier survival analysis and compared using the log-rank test. Spearman’s correlation analysis was performed to determine the correlation between variables. Statistical significance was set at *p* < 0.05.

### Supplementary information

supplementary information

Original Data File

## Data Availability

The data used or analyzed during this study are included in this article and available upon request by contact with the corresponding author.
