# Supplementary material for: Nucleophosmin 1 cooperates with BRD4 to facilitate c-Myc transcription to promote prostate cancer progression
Source: Cell Death Discov. 2023 Oct 24;9:392. doi: 10.1038/s41420-023-01682-w (PMC10597990; doi:10.1038/s41420-023-01682-w)
Supplement: Supplementary file 1 — supplementary information [file 41420_2023_1682_MOESM1_ESM.pdf]

Supplementary Fig. S1 Legend

The correlations between NPM1 and MYC mRNA expressions in breast adenocarcinoma, pancreatic adenocarcinoma, stomach adenocarcinoma, lung carcinoma, thyroid carcinoma, colon carcinoma and cervical carcinoma were determined by the GEPIA web tool. The *p* and R values are shown as indicated.

Supplementary Fig. S1

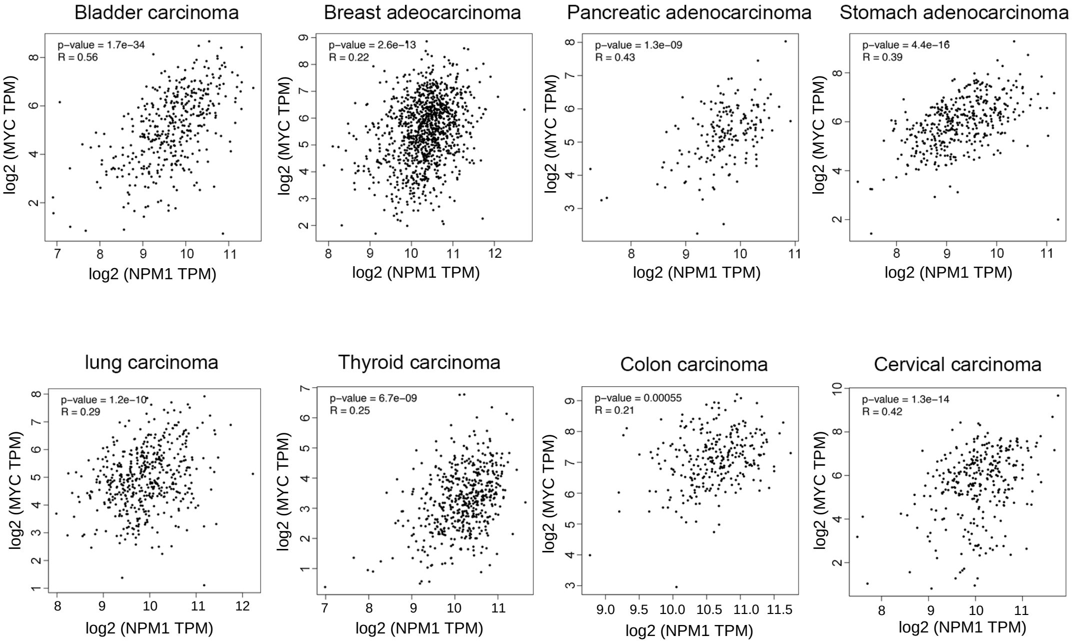

Supplementary Table S1.

Antibodies

Antibody	Source	Identifier
NPM1	Abcam	Ab10530
CAPDH	Cell Signaling Technology	#92310
β-Tubulin	Cell Signaling Technology	#5666
c-Myc	Cell Signaling Technology	#18583
HA-Tag	Cell Signaling Technology	#48823
BRD4	Abcam	ab75898
Goat anti-rabbit IgG-HRP	Beyotime	A0208
Goat anti-mouse IgG-HRP	Beyotime	A0216
