# Supplementary material for: Nucleophosmin 1 cooperates with BRD4 to facilitate c-Myc transcription to promote prostate cancer progression
Source: Cell Death Discov. 2023 Oct 24;9:392. doi: 10.1038/s41420-023-01682-w (PMC10597990; doi:10.1038/s41420-023-01682-w)

Original Data Figure 1

B

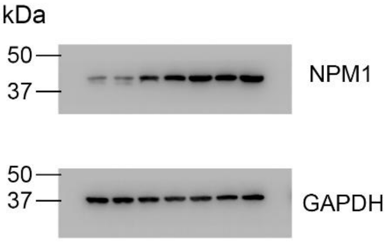

F

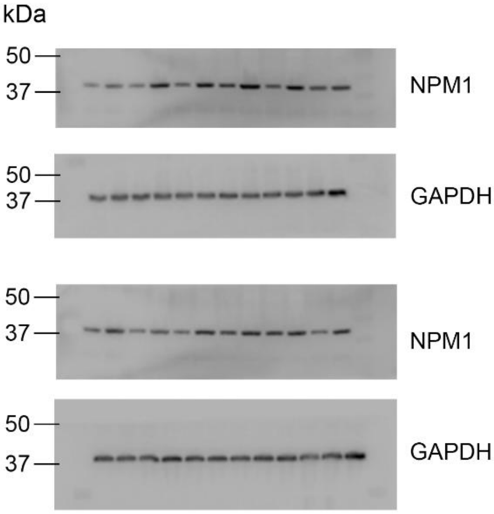

Original Data Figure 2

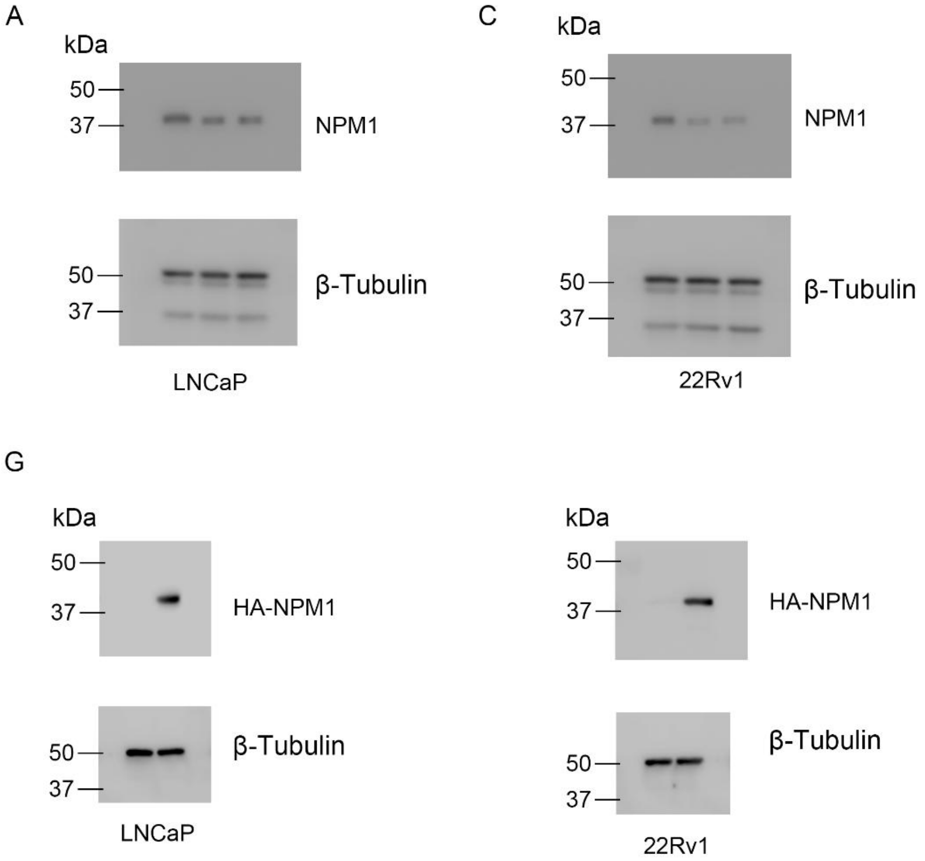

Original Data Figure 3

A

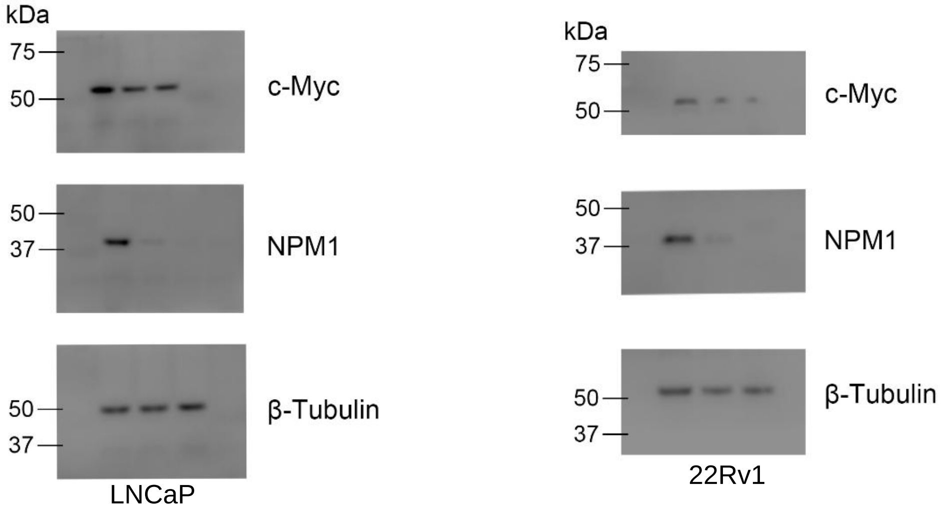

D

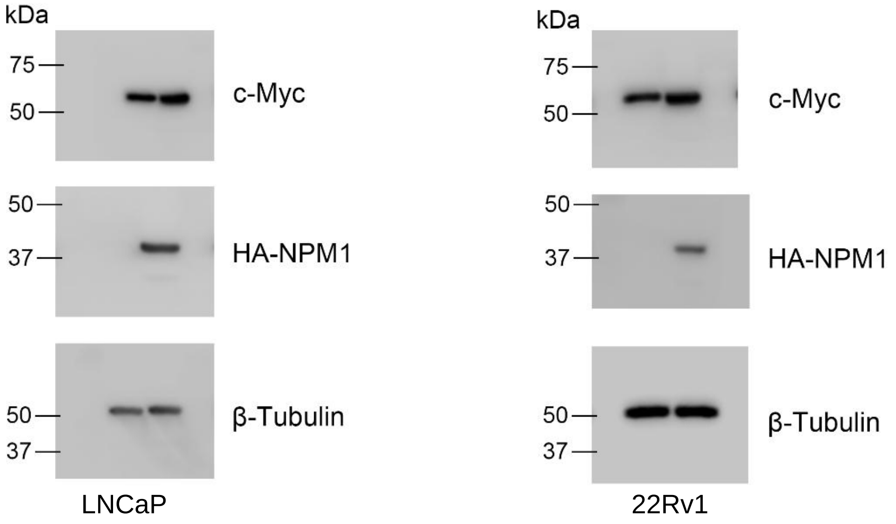

Original Data Figure 4

A

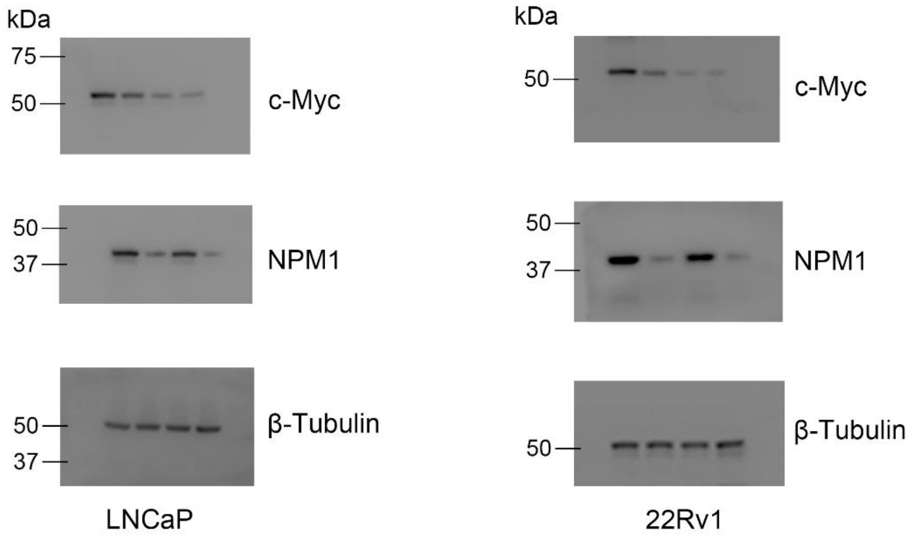

Original Data Figure 5

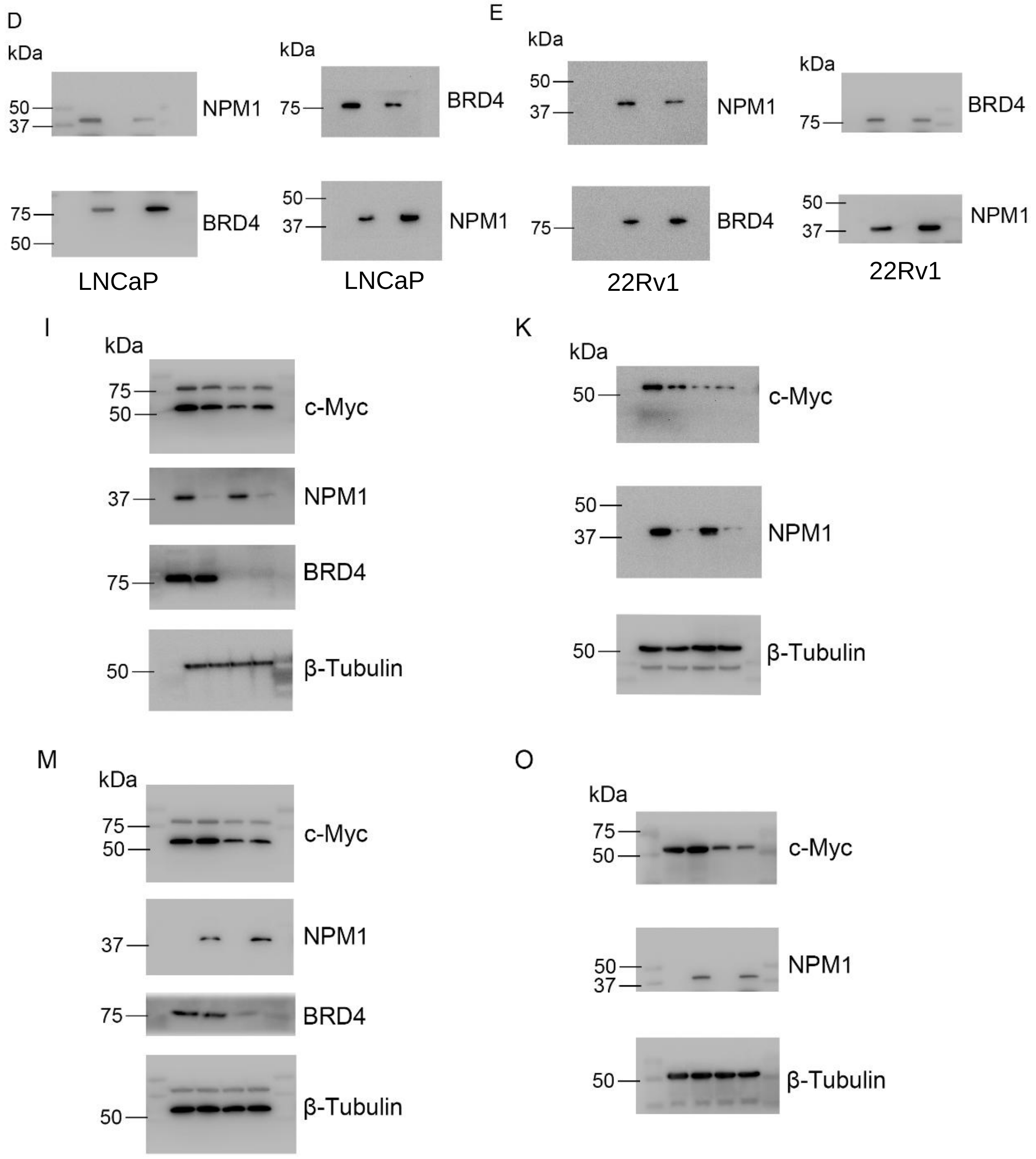

Original Data Figure 6

A

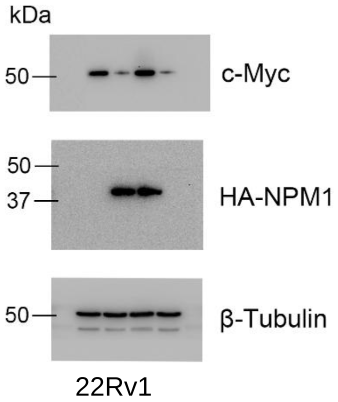

Supplement: Supplementary file 2 — Original Data File [file 41420_2023_1682_MOESM2_ESM.pdf]
